# The Isorhamnetin-Containing Fraction of Philippine Honey Produced by the Stingless Bee *Tetragonula biroi* Is an Antibiotic against Multidrug-Resistant *Staphylococcus aureus*

**DOI:** 10.3390/molecules26061688

**Published:** 2021-03-17

**Authors:** Angelica Faith L. Suarez, April Dawn G. Tirador, Zenith M. Villorente, Cathrina F. Bagarinao, Jan Vincent N. Sollesta, Gerard G. Dumancas, Zhe Sun, Zhao Qi Zhan, Jonel P. Saludes, Doralyn S. Dalisay

**Affiliations:** 1 Center for Chemical Biology and Biotechnology (C2B2), University of San Agustin, Iloilo City 5000, Philippines; aicasuarez03@gmail.com (A.F.L.S.); tiradorapril@gmail.com (A.D.G.T.); 2 Maridan Industries, Inc., Jaro, Iloilo City 5000, Philippines; zmvillorente@maridan.com.ph (Z.M.V.); cfbagarinao@maridan.com.ph (C.F.B.); jvsollesta@maridan.com.ph (J.V.N.S.); 3 Department of Mathematics and Physical Sciences, Louisiana State University at Alexandria, Alexandria, LA 71302, USA; gdumancas@lsua.edu; 4 Balik Scientist Program, Philippine Council for Health Research and Development (PCHRD), Department of Science and Technology, Bicutan, Taguig City 1631, Philippines; jsaludes@usa.edu.ph; 5 Shimadzu Asia Pacific (SAP), Singapore Science Park I, Singapore 118264, Singapore; sunzhe@shimadzu.com.sg (Z.S.); zhaoqi@shimadzu.com.sg (Z.Q.Z.); 6 Center for Natural Drug Discovery and Development (CND3), University of San Agustin, Iloilo City 5000, Philippines; 7 Department of Chemistry, College of Liberal Arts, Sciences, and Education, University of San Agustin, Iloilo City 5000, Philippines; 8 Department of Biology, College of Liberal Arts, Sciences, and Education, University of San Agustin, Iloilo City 5000, Philippines

**Keywords:** Philippine honey, *Tetragonula biroi*, stingless bee, flavonoids, antibacterial, antioxidant, isorhamnetin, phenolics

## Abstract

Honey exhibits antibacterial and antioxidant activities that are ascribed to its diverse secondary metabolites. In the Philippines, the antibacterial and antioxidant activities, as well as the bioactive metabolite contents of the honey, have not been thoroughly described. In this report, we investigated the in vitro antibacterial and antioxidant activities of honey from *Apis mellifera* and *Tetragonula biroi*, identified the compound responsible for the antibacterial activity, and compared the observed bioactivities and metabolite profiles to that of Manuka honey, which is recognized for its antibacterial and antioxidant properties. The secondary metabolite contents of honey were extracted using a nonionic polymeric resin followed by antibacterial and antioxidant assays, and then spectroscopic analyses of the phenolic and flavonoid contents. Results showed that honey extracts produced by *T. biroi* exhibits antibiotic activity against *Staphylococcal* pathogens as well as high antioxidant activity, which are correlated to its high flavonoid and phenolic content as compared to honey produced by *A. mellifera*. The bioassay-guided fractionation paired with Liquid Chromatography Mass Spectrometry (LCMS) and tandem MS analyses found the presence of the flavonoid isorhamnetin (3-methylquercetin) in *T. biroi* honey extract, which was demonstrated as one of the compounds with inhibitory activity against multidrug-resistant *Staphylococcus aureus* ATCC BAA-44. Our findings suggest that Philippine honey produced by *T. biroi* is a potential nutraceutical that possesses antibiotic and antioxidant activities.

## 1. Introduction

The health-promoting properties of honey are associated with plant secondary metabolites that are gathered by honey bees concomitant to the collection of flower nectars or honeydew. In addition to its traditional household use as a sweetener, honey is often recognized as an alternative nutraceutical that exhibits a broad spectrum of in vitro activities such as antibacterial, antioxidant, anti-inflammatory, immunomodulatory, anticancer, anti-glycemic, prebiotic, and antiviral activities [1,2,3,4,5,6,7,8]. Correlated with honey’s biological activities is the diversity of its secondary metabolite composition. This chemodiversity is influenced by several factors, such as plant source, geographical location, entomological origin, honey collection, and extraction processes [8,9,10,11,12,13]. Aside from being a supersaturated solution of glucose and fructose, the chemical composition of honey is complex and variable, which is strongly dependent on its geographical and botanical origins [14]. Estimates indicate that honey is composed of about 200 other minor metabolites that are usually present between 0.01 and 10 ppm [15]. Honey contains plant-based polyphenols, aromatic acids, esters, and flavonoids that are vital for honey’s antioxidant and antibacterial properties [16,17,18,19,20,21,22]. Aside from phenolic compounds, honey also contains enzymes such as glucose oxidase, diastase, invertase, catalase, and peroxidase that are also responsible for antibacterial activity [1,2,6]. Other bioactive constituents such as organic acids, trace elements, vitamins, amino acids, and proteins were also found [1,2,19].

An abundance of information on the evaluation of honey’s antimicrobial and antioxidant properties is available [3,7,8,16,20,23]. However, it is notable that the observed bioactivities are dependent on and as variable as the botanical and geographical origins, and climatic conditions [8,9,10,11,13,14,23]. In the Philippines, the majority of honey is produced by *A. mellifera* and, to a limited extent, by the indigenous stingless bee *Tetrogonula biroi* (synonymous to *Trigona biroi*) [24]. Reports showed that honeys produced by the stingless bee *T. biroi* were produced in Southern Luzon area in the Philippines, particularly in the Laguna, Quezon, and Sorsogon regions, and Western Visayas area specifically in Iloilo and Negros Occidental provinces [24,25,26,27]. Conversely, *A. mellifera* honey is produced in the Southern Mindanao (Davao) [28] and Southern Luzon areas in Laguna [29]. These local honeys are used as sweeteners, but their potential as nutraceutical products with health and therapeutic benefits has not been thoroughly described. As part of our bioactive natural products program, we performed a small-scale screening of Philippine honeys samples produced by *A. mellifera* and *T. biroi* for their antibacterial and antioxidant activities, and determined their total phenolics and total flavonoid contents. We used Manuka honey, a widely recognized nutraceutical product for its antibacterial and antioxidant properties, as a reference honey [30,31]. Lastly, we identified an antibiotic compound from the honey produced by Philippine stingless bee *T. biroi* that possesses high antibacterial activity against *S. aureus* ATCC BAA-44, a multidrug resistant strain. The overall findings of our study strongly support and validate that Philippine honey produced by the stingless bee *T. biroi* is a nutraceutical with demonstrated in vitro therapeutic potential.

## 2. Results

### 2.1. Antibacterial Activities

The honey crude extracts isolated from 15 local apiaries in the Philippines (Table 1, Figure S1) were tested against five target *Staphylococcus* pathogens, namely *S. aureus* ATCC 25923, *S. aureus* ATCC BAA-44, *S. aureus* ATCC 6538, *S. saprophyticus* clinical isolate, and *S. epidermidis* clinical isolate using agar well diffusion assay. The antibacterial assay results showed that Philippine honey SL 01 crude extract produced by the stingless bee *T. biroi* showed inhibitory activity against all five tested pathogens (Table 2). It exhibited the highest activity with 12.5 mm zone of inhibition (ZOI) against *S. aureus* ATCC BAA-44, a multidrug-resistant pathogen. It showed minimal activities (3.5 to 5.0 mm ZOI) against the remaining *Staphylococcus* pathogens. The SL 01 crude extract was the only sample (7% hit rate) with activity against *S. aureus* ATCC BAA-44, *S. aureus* ATCC 25923, and *S. epidermidis* clinical isolate (Table 2). There were four (27% hit rate) honey extracts that showed activity against *S. aureus* ATCC 6538, namely NM 01, SL 01, SEM 01, and WV 01, with weak activity of 1.5 to 3.5 mm ZOI. Twelve (80% hit rate) extracts showed activity (ZOI 2.5 to 5.0 mm) against *S. saprophyticus*. NL 02 and WV 02 honey extracts were found to be inactive against all five tested pathogens. Interestingly, none of the Manuka honey crude extracts showed antibacterial activities against all five tested pathogens and showed only minimal activity (ZOI 2.0 to 2.5 mm) against the *S. aureus* ATCC BAA-44. Notably, SL 01 honey extract produced by the stingless bee *T. biroi* possessed antibiotic activity against all five tested *Staphylococcal* pathogens, while the remaining honey samples produced by *A. mellifera* showed selective activity against the tested pathogens (Table 2).

### 2.2. Antioxidant Activities

**Figure 1:**
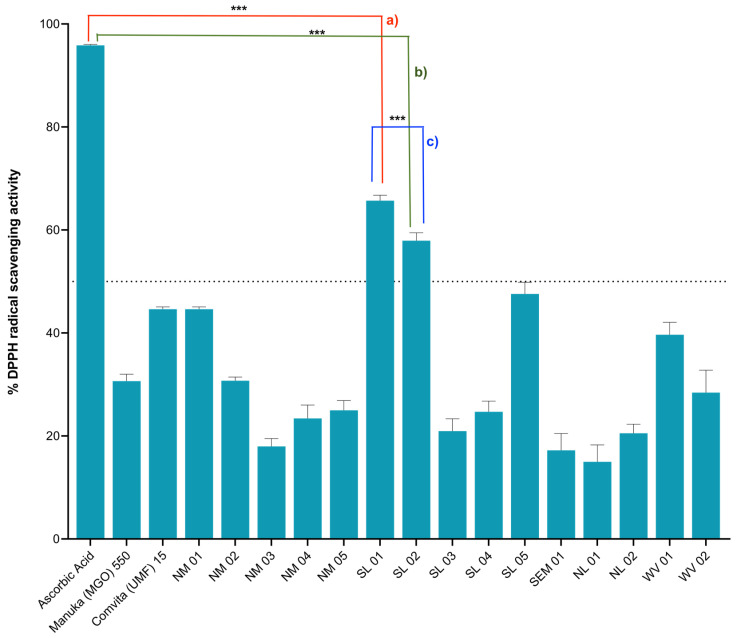
2,2-diphenyl-1-picrylhydrazyl (DPPH) Scavenging Activity of Honey Crude Extracts. Ascorbic acid, 1 mg/mL (positive control); Honey extracts, 5 mg/mL. (**a**) Two tailed *p* value = 0.0008 for ascorbic acid and SL01, (**b**) Two tailed *p* value = 0.0013 for ascorbic acid and SL02, (**c**) Two tailed *p* value = 0.0040 for SL01 and SL02. *** indicate the significant difference (*p* ≤ 0.05).

To evaluate if Philippine honey crude extracts possess antioxidant activities, 2,2-diphenyl-1-picrylhydrazyl (DPPH) radical scavenging assay was performed. Among the honey extracts tested for antioxidant activity, SL 01 produced by the stingless bee *T. biroi* exhibited the highest activity at 65.71% inhibition, followed by SL 02 produced by *A. mellifera*, with 57.94% inhibition. There was a significant difference between SL 01 and SL 02 honey extracts antioxidant activities as shown by *p* ≤ 0.05. The rest of the Philippine honey extracts showed minimal antioxidant activity (<50%) when referred to the positive control, ascorbic acid (Figure 1). Interestingly, the Manuka honey crude extracts showed minimal activity as well at <50% antioxidant activity.

### 2.3. Flavonoid and Phenolic Contents and their Correlation with Bioactivities

The phenolic and flavonoid contents of honey extracts (Table 3) were quantified using spectroscopic techniques. Our findings revealed that Philippine honey extracts contain phenolics at a concentration range of 236–165 μg Gallic Acid Equivalent (GAE)/mg crude extract. Philippine honeys with high phenolic contents were NM 02, NM 05, SL 05, NL 02, SL 04, and SL 01 samples (Table 3). Interestingly, the SL 01 honey from stingless bee *T. biroi* showed the highest flavonoid content at 216.14 μg Quercetin Equivalent (QE)/mg (Table 3) and the remaining honey samples showed low flavonoid content with <30 μg QE/mg. Conversely, Manuka honey extracts showed low levels of phenolic (184–170 μg GAE/mg) and flavonoid (21–26 μg QE/mg) contents.

**Figure 2:**
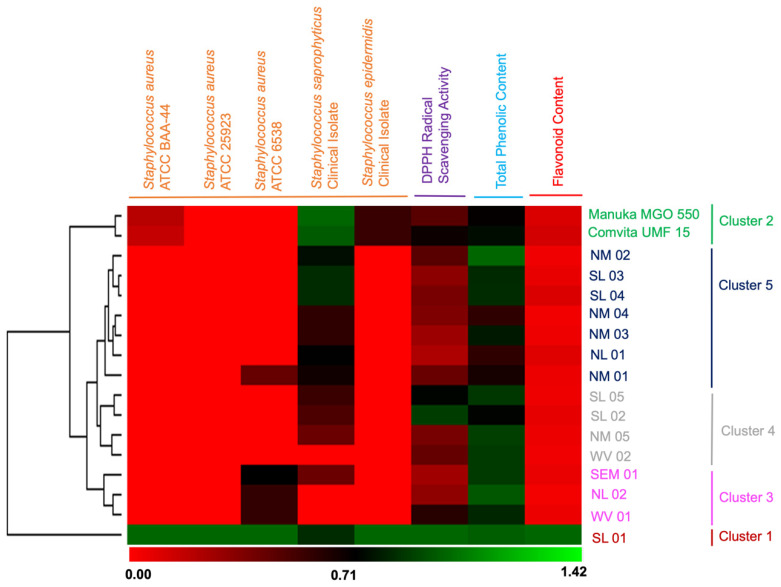
Heat map and hierarchical clustering presentation of antibacterial activity, antioxidant activity, total phenolic content, and flavonoid content of honey crude extract samples.

Hierarchical clustering was constructed to correlate the flavonoids and phenolics contents in honey extracts with its observed antibacterial and antioxidant activities (Figure 2). The between-groups linkage method was chosen as a complete linkage and the City Block Distance was selected to establish clusters. A dendrogram provided the visual representation of the relationship of bioactivities as affected by total phenolic content and flavonoid content. The resulting hierarchical cluster heat-map in Figure 2 produced well-defined clusters that were grouped into five main clusters. Honey SL 01 (stingless bee *T. biroi*) was the only sample categorized in cluster 1 with a longer distance of similarity to the four clusters. Cluster 1 showed activity against all five tested pathogens, with the highest antioxidant activity, a high total phenolic content (TPC), and the highest flavonoid content. To note, SL 01 honey bees (*T. biroi*) forage on coconut trees, bananas, and mangoes (Table 1). The Manuka honey samples (MGO 550 and UMF 15) were grouped together into cluster 2, indicating similarity in bioactivity and phenolics and flavonoid contents. The *A. mellifera* producing this honey forages on the manuka tree (Table 1). The cluster 3 was composed of NL 02, WV 01, and SEM 01. These samples possess antibacterial activities towards two (WV 01) or three (SEM 01) target tested pathogens and moderate antioxidant activities (Figure 2). Interestingly, NL 02 showing no antibacterial activities was included in this cluster. The floral sources for this cluster are various plants such as bananas, coconuts, sunflowers, trumpet flowers, and squash (Table 1). Honey samples WV 02, NM 05, SL 02, and SL 05 were categorized in cluster 4. These samples possess moderate activity against *S. saprophyticus*, weaker antioxidant activity, and a high phenolic content (between 235 and 220 μg GAE/mg sample). Members in cluster 4 are honey samples with bees foraging in various plants such as pineapples, coconuts, mangroves, trumpet flowers, and kerson fruit (Table 1). The remaining samples with the weakest biological activities and the least flavonoid contents were grouped in cluster 5. Four of these samples (NM 01, NM 02, NM 03, NM 04, and NL 01) were produced by *A. mellifera* bees foraging either in pineapples or, sunflower (Table 1). Samples SL 03 and SL 04 were honeys made by *A. mellifera* from various floral sources.

### 2.4. Purification of the Antibiotic Component from T. biroi Honey

Since *T. biroi* honey extract (SL 01) inhibited the growth of all the tested organisms, including the multidrug-resistant *S. aureus* ATCC BAA-44 with Minimum Inhibitory Concentration, MIC at 1250 μg/mL, it was purified by Gel Permeation Chromatography (GPC) using Sephadex LH-20 and yielded 69 fractions of 7 mL each that were then pooled to eight fractions (Table 4). The bulk of the crude extract was found in Fraction 4 with 219.2 mg, 42.98% yield. Subsequently, these eight pooled GPC fractions were tested for antibiotic activity against multidrug-resistant *S. aureus* ATCC BAA-44. Fractions 4 and 7 were found to inhibit the tested pathogen at 72.5% and 82.1%, respectively. There was no significant difference found in the antibiotic activities of Fractions 4 and 7, as shown by *p* value of 0.4109 using two-tailed test (*p* ≤ 0.05) (Table 4). The remaining GPC fractions were inactive against the tested pathogen.

**Figure 3:**
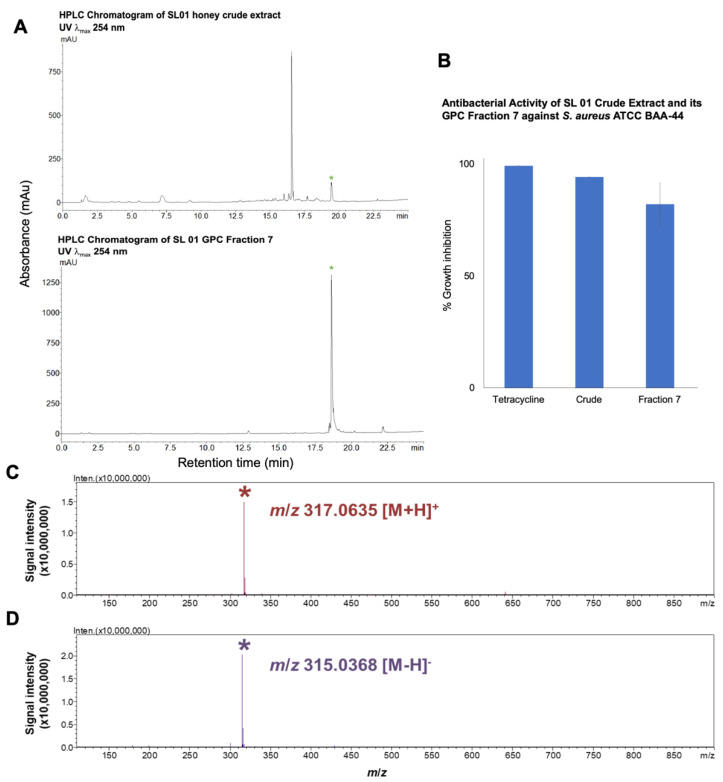
(**A**) High performance liquid chromatography (HPLC) Chromatogram of SL 01 crude extract and GPC fraction 7 showing a peak at λ_max_ 254 nm that eluted at 18.7 min. (**B**) Antibacterial activities of SL 01 honey crude extract and Fraction 7 against *S. aureus* ATCC BAA-44 at 5 mg/mL; Tetracycline at 0.25 mg/mL as positive control. DMSO showed no inhibitory activity towards *S. aureus* ATCC BAA-44. LCMS-IT-TOF analysis of peak at 18.7 min showing a measured mass at (**C**) *m*/*z* 317.0635 [M + H]^+^ and (**D**) *m*/*z* 315.0368 [M − H]^−^.

For high performance liquid chromatography (HPLC) profiling of the GPC fractions, we focused on Fraction 7 because the HPLC chromatogram revealed a single peak at λ_max_ 254 nm that eluted at 18.7 min (Figure 3A) and showed 82.1% inhibitory against *S. aureus* ATCC BAA-44 (Figure 3B). The liquid chromatography mass spectrometry-ion trap-time of flight (LCMS-IT-TOF) analysis of this peak showed a compound with mass at *m*/*z* 317.0635 [M + H]^+^ and *m*/*z* 315.0368 [M + H]^−^ (Figure 3C,D). MS/MS analysis revealed that the compound is the flavonoid isorhamnetin (Figure S5), whose identity was unambiguously confirmed by the fragment ions at *m*/*z* 302 ([(M + H) − 15]^+^) and 285 ([(M + H) − (15 + 17)]^+^) due to the fragmentation of the methyl at 3′ position and -OH at 7 position. The mass loss of the 165 m.u. to produce a fragment ion *m*/*z* 153 showed that the additional methyl was linked to this fragment at 3′ position instead of at 7 position (Figure S5). Further, the MS/MS fragmentation of this bioactive compound matches that of isorhamnetin in the MS/MS Spectrum Match program in METLIN^TM^ Database [32] (Figure S5). There is a likelihood that the prominent UV peak at 16.7 min observed in crude extract (Figure 3A) should it corresponds to an analogue of isorhamnetin could provide insight on structure-activity relationship. However, the UV peak at 16.7 min went to inactive Fraction 5 during fractionation by GPC (Table 4 and Figure S6). The bioactive constituents in Fraction 4 as shown by numerous UV peaks at λ_max_ 254 nm (Table 4 and Figure S7), warrant further research, which thus constitutes part of our continuing natural products discovery program.

## 3. Discussion

Herein we report that secondary metabolites present in honey possess antibacterial and antioxidant activities. Our findings revealed that the selected Philippine honey extracts in this study showed in vitro antibacterial activities against *Staphylococcal* pathogens. The antibacterial activity, however, varied with the honey type. It was shown that honey extract produced by the *A. mellifera* bee species exhibits weak antibiotic activities, while the SL 01 honey extract produced by *T. biroi* exhibits moderate to high antibacterial activity against all *Staphylococcal* pathogens tested, including the multidrug-resistant *S. aureus* ATCC BAA-44. Nonetheless, it is evident that *T. biroi* honey extract possesses higher and broader antibacterial activities compared to *A. mellifera* honey extracts. This finding corroborates the report that the stingless bee honey (SBH) demonstrates superior antibacterial activities when compared to European bee honey (EBH) produced by *Apis* spp [33,34,35].

Honey has several well-known characteristics that are generally accepted as contributing factors in the overall antimicrobial activity, which include low pH, an osmotic effect, hydrogen peroxide production, and phytochemical factors [1,2]. In this study, *T. biroi* (SL 01) honey extract remained active against five *Staphylococcal* pathogens after the removal of oligosaccharides and moisture, thus, indicating that the antibacterial component is non-volatile and non-peroxidase in contrast to other studies in honey whereby antibacterial activity is attributed to the presence of enzymes such as catalase and peroxidase [1,2,6]. There were several UV peaks at λ_max_ 254 nm that may contribute to the antibiotic activity of SL 01 extract Fraction 7. Nevertheless, the isorhamnetin (3-methylquercetin), a plant flavonoid that we detected in Fraction 7, was the major UV peak (λ_max_ 254 nm) that plausibly contributed to the nonperoxide antibacterial activity of the *T. biroi* honey. A report on the mechanism of action of methylated flavonoids such as isorhamnetin showed that it targets bacterial cell membrane by increasing permeability, leading to cessation of ATP synthesis capacity, membrane transport, and motility [36]. It has been demonstrated that isorhamnetin inhibits the growth of *S. aureus* by down-regulating its RNAIII expression and inhibiting the alpha-hemolysin (Hla) transcription [37]. This metabolite, together with other flavonoids, may exhibit its antibacterial activity by cell lysis and disruption of the cytoplasmic membrane upon permeability [37,38,39].

The antibacterial activities of Manuka honey crude extract in this study do not agree with the reported potent bioactivity of methylglyoxal (MGO) [31,40]. Perhaps the solid-phase extraction and in vacuo drying processes (35–40 °C) removed the volatile MGO. Nonetheless, our findings concur with previous reports that MGO is not solely responsible for Manuka honey’s antimicrobial activity [30,41,42].

We demonstrated that SL 01 honey extract possesses the highest antioxidant activity among the selected Philippine honey extract tested, and whose activity attribute to its high phenolic and flavonoid contents. This finding corroborates with a recent study on *T. biroi* honey, which claims that phenolic acids and flavonoids are responsible for the well-established antioxidant activity of stingless bee honey [25]. There are reports indicating that phenolics and flavonoids in honey are greatly influenced by the floral source where the bee species forage for food [8,14,43]. In this preliminary study, the correlation of antibiotic and antioxidant activities to the botanical and entomological origins of honey is not clear because of narrow set of samples and lack of melissopalynological analysis information. Nonetheless, the results of the study are promising, as we have demonstrated the potential antibacterial and antioxidant activities of Philippine honeys derived from *A. mellifera* and stingless bee *T. biroi*. For some honey extracts with high phenolic content such as NM 02, NM 05, and SL 05, the amount of phenolic content was not directly correlated to its antioxidant activity. This observation could be due to some other constituents that suppress its radical scavenging activity, as the examined samples were from crude extracts.

The clustering analysis suggests that bioactivities (antibiotic and antioxidant) correlate directly to the total phenolic content and flavonoid contents as demonstrated by SL 01 honey extract produced by a stingless bee *T. biroi,* followed by the Manuka honey extracts in cluster 2. It is clear that the well-defined clustering as displayed in the dendrogram is consistent with the direct correlation between bioactivities and phenolic and flavonoid contents, i.e., honey extracts with a low amount of phenols and flavonoids, consequently, showed weak antibacterial activity.

In this work, the *T. biroi* honey crude extract showed the highest content of phenolics and flavonoids, with isorhamnetin as one of its antibiotic components. Isorhamnetin is known to be present in various plants such as mangoes and bananas [44,45,46], which suggests the possible origin of isorhamnetin in *T. biroi* honey, since the foraging area of the bee hives was surrounded by coconut, banana, and mangoes trees. Interestingly, there were also honeys in this study produced by *A. mellifera* (WV 02, SEM 01, and SL 04) that foraged in mangoes and bananas, yet showed minimal antibiotic activities. Although there are limited studies on the foraging mechanisms of *T. biroi*, it was suggested that its smaller body size allows itself to gather food from the different parts of the floral source, resulting in high flavonoids and phenolic acid content [47]. Another plausible explanation for the source of isorhamnetin found in *T. biroi* honey is the cerumen, also known as stingless bee propolis [48]. The cerumen is a mixture of wax and plant resins that are used as material in building the stingless bee nest. Plant resins are rich source of flavonoids and are well-known for their potent antimicrobial properties [49]. Unlike *A. mellifera* that preserves honey by a dehydration technique while being stored in the honeycomb, the stingless bee (e.g., *T. biroi*) relies on intense fermentation by symbiont microbes and by aging inside the cerumen pot to preserve the honey [50]. While aging, the aromas from the cerumen are incorporated into honey, giving its intense aroma compared to *A. mellifera* honey. It has been reported that cerumen extracts have anti-inflammatory and antimicrobial properties [49,51,52] and it is possible that the antibiotics and antioxidants from the cerumen are integrated in the honey during aging process [50]. Report showed that a total of 100 compounds have been identified from stingless bee propolis from 2000 to 2019 by groups in Brazil, South Asia and Australia [48]. To note, isorhamnetin was isolated from the nest of stingless bee *T. spinipes* collected in Fortaleza, state of Ceará, Northeast of Brazil [53]. Collectively, the findings of this study on the anti-staphylococcal activity of *T. biroi* honey extract and reports on the biological potential of stingless bee honey from Brazil [49,54], South Asia [25,26,47,55,56], and Australia [48,51,57] underscore the beneficial properties and nutritional value of stingless bee honey.

## 4. Materials and Methods

### 4.1. Honey Samples

A total of 15 raw honey samples produced by *A. mellifera* (14 samples) and stingless bee *T. biroi*, (1 sample) were collected from different apiaries in the Philippines on September to December 2016. All honey samples were obtained from a local bee farm. In lieu of melissopalynological analysis to determine the floral and botanical origins of honey samples, the identification of botanical origins was performed based on the geographical foraging area and floral availability where bee hives were located. Only one *T. biroi* honey was collected during that period because of limited production. For *A. mellifera* honey samples, two were collected from Northern Luzon (NL), five were from Southern Luzon (SL), two were from Western Visayas (WV), one from Southeastern Mindanao (SEM), and five from Northern Mindanao (nm) (Table 1 and Figure S1). Two types of Manuka honey: Comvita™ (UMF 15), and Manuka Health™ (MGO 550) were used as references due to their standardized methylglyoxal (MGO) content that was claimed to be antibacterial.

### 4.2. Reagents and Standards

The Amberlite™ XAD-16N resin, tetracycline, 2,2-diphenyl-1-picrylhydrazyl (DPPH), ascorbic acid, and quercetin (≥95%) were obtained from Sigma-Aldrich (St. Louis, MO, USA). The gallic acid (99.3%) was obtained from ChromaDex (Los Angeles, CA, USA). Acetonitrile, methanol, ethyl acetate, water, and formic acid (LC-MS grade) were also purchased from Sigma-Aldrich (St. Louis, MO, USA).

### 4.3. Metabolite Extraction

Solid Phase Extraction (SPE) method was utilized to extract the secondary metabolite contents of honey [58]. Briefly, 25 g of honey samples were dissolved in 75 mL acid water (adjusted to pH 2.0). The solution was treated with 37.5 g Amberlite™ XAD-16N resin (100 μm) and gently stirred for 30 min to allow adsorption of metabolites. After stirring, the resin was washed with 2 L acid water followed by 1 L distilled water. Subsequently, the resin was washed with methanol (750 mL) and ethyl acetate (750 mL) to extract the adsorbed metabolites. This step was repeated three to four times until the resin returned to its original white appearance. The combined methanol and ethyl acetate extracts (9–10 g, 36–40% yield) were concentrated in vacuo at 35 to 40 °C, lyophilized, and stored at −80 °C until used for in vitro antibacterial and antioxidant assays and chemical profiling.

### 4.4. Target Organisms for Antibacterial Testing

The target bacterial pathogens used in this study were *Staphylococcus aureus* ATCC 25923, *S. aureus* ATCC BAA-44 (multidrug-resistant), *S. aureus* ATCC 6538, *S. saprophyticus* clinical isolate, and *S. epidermidis* clinical isolate. These test organisms were grown and maintained at 37 °C in tryptic soy agar for no more than 24 h until they were used for antibacterial assay.

### 4.5. Agar Well Diffusion for Antibacterial Assay

The honey extracts obtained from SPE were tested for antibacterial activity using the agar well diffusion method. Briefly, ten mL of bacterial cell suspension was adjusted to obtain an optical density (OD) of 1 × 10^6^ CFU/mL and mixed thoroughly with 40 mL of 1.5% Mueller-Hinton Agar (MHA) at 40 °C. MHA inoculated with bacterial cells was poured into a 150 mm Petri dish and allowed to solidify. After solidification, agar wells were made using sterile borer to make a 6 mm well. Honey extracts in dimethyl sulfoxide (DMSO) were prepared at 200 mg/mL stock solution. Fifty μL of honey extracts was dispensed into each well corresponding to a treatment of 10 mg sample per well. Fifty μL of tetracycline (5 mg/mL in DMSO) and 50 μL DMSO were dispensed into separate wells as positive and negative controls, respectively. All test pathogens in this study were not sensitive to DMSO (Table 2). The plates were incubated at 37 °C for 18 h followed by measurement of the zone of inhibition (ZOI, mm) after subtracting the 6 mm diameter of the well. All tests were performed in duplicate.

### 4.6. Antioxidant Assay Using 2,2-Diphenyl-1-Picrylhydrazyl (DPPH)

To provide a preliminary screening for the antioxidant activity of the honey extracts, the radical scavenging activity was assessed using the free radical DPPH assay, as described previously [59] with few modifications. Twenty μL of each honey extracts (50 mg/mL in DMSO, pH 3.5 to 4.5) and 180 μL of DPPH (Sigma Aldrich, St. Louis, MO, USA) solution (300 μM in methanol) were dispensed in wells of a 96-well plate. Twenty μL of DMSO with 180 μL DPPH solution was used as the negative control, while ascorbic acid (Sigma Aldrich, (St. Louis, MO, USA) (10 mg/mL) was used as a positive control. The plate was incubated in the dark at room temperature for 30 min followed by the measurement of absorbance at 570 nm using the microplate plate reader (Multiskan FC, Thermo Scientific, Torrance, CA, USA). The assay was performed in triplicates. The % DPPH radical scavenging activity of honey extracts was determined using the following formula:

% DPPH radical scavenging activity = (Absorbance of negative control − Absorbance of the sample)/Absorbance of negative control) × 100(1)

### 4.7. Quantification of Flavonoids

The flavonoid content of honey was determined through the formation of aluminum-flavonoid complexes, with some modifications [59]. An aliquot of AlCl_3_ solution (4 μL, 10% *w*/*v*) was added to honey extract aqueous solution (20 μL, 50 mg/mL) and subsequently treated with H_2_O (112 μL), 60 μL methanol and 1 M CH_3_COONa (4 μL). Quercetin (≥95%, Sigma Aldrich, St. Louis, MO, USA) was used as standard at a concentration range of 0 to 100 μg/mL to establish the calibration curve. The mixture was vigorously shaken and incubated at 24 °C in a microplate reader (BMG Labtech, Offenburg, Germany) for 10 min. The absorbance was read at 415 nm. The assay was performed in triplicates.

### 4.8. Determination of Total Phenolic Content

The Folin-Ciocalteu (F-C) assay while traditionally used for total phenolic content (TPC) in plant food extracts may produce inaccurate estimations of TPC values due to the presence of reducing interferants such as ascorbic acid [60]. In order to mitigate this limitation, we utilized a more novel method using chemometrics (i.e., partial least squares) and FTIR. PLS offers the advantages of simplicity and being able to quantify analytes of interest from a complex matrix [61,62,63]. The total phenolics content (TPC) of honey extract solution (10 μL, 500 mg/mL in methanol) was quantified by Attenuated Total Reflectance-Fourier Transform Infrared Spectroscopy (ATR-FTIR) [64]. FTIR spectra were recorded at room temperature (22 °C) using IRAffinity-1S spectrometer (Shimadzu, Kyoto, Japan) scanning through the frequency range of 4000 to 700 cm^−1^ at a resolution of 2 cm^−1^. The final IR spectrum of each honey extract was an average of 128 scans with two spectra taken per aliquot. The experiment was performed in triplicates.

The IR spectra of honey extracts averaged and were processed using the R software (R Development Core Team, Vienna, Austria) for further analysis. Savitkzy Golay was performed, with a three-point filter and wavelet transform scale of three to enhance the resolution of spectral features and to minimize problems from baseline shifts. Partial least-squares regression (PLSR) with three components was employed for the quantitative analysis. A total of seven spectra of gallic acid (99.3%, Chromadex, Los Angeles, CA, USA) were used to establish the calibration model. A leave-one-out cross validation was performed to evaluate the accuracy of the model by removing one standard from the data set at a time and applying a calibration to the remaining standards (Figures S2–S4).

### 4.9. Purification and Isolation of Antibiotic Compound from Philippine Honey

The crude honey extract obtained was purified by gel permeation chromatography (GPC). Crude extract (510.0 mg) was dissolved in 1.0 mL methanol and loaded to a 3.3 cm × 35 cm column Sephadex^®^ LH-20 (bead size: 25–100 μm, bed volume: 307.9075 cm^3^, linear flow rate: 9.82 cm/h). The column was eluted with methanol and 69 of 7 mL fractions were collected. We attempted to pool the eluates according to TLC profile, however the compounds present in the eluate did not resolve well in silica plate. Consequently, eluates were pooled into 8 major fractions based on their visible color profiles, the pooled fractions were concentrated in vacuo, and freeze dried for subsequent assays and chemical profiling analyses.

### 4.10. Chemical Profiling of Bioactive Honey GPC Fraction Using High Performance Liquid Chromatography (HPLC)-Diode-Array Detector (DAD)

The GPC fractions were profiled using HPLC (LC-20AD Prominence and PDA SPDM20A Prominence, Shimadzu, Kyoto, Japan). Briefly, GPC fractions at 10 mg/mL (MeOH:H_2_O, 1:1) were filtered using a 0.2 μm filter syringe. Twenty μL of the filtered GPC solution was injected in HPLC. The purification was carried out on a reversed phase column (Phenomenex Kinetex C_8_ column, 2.6 μm, 100 × 4.60 mm) using a low-pressure gradient elution solvent system (solvent A: water with 0.1% TFA, solvent B: methanol with 0.1% TFA) at a flow rate of 0.8 mL/min. The gradient started with 95% solvent A at 0.01 to 5 min, 60% solvent A at 10 min, 30% solvent A at 15 min, 0% solvent A at 20 to 33 min, back to 95% solvent A at 35 min. The UV peaks (λ_max_ 254 nm) of the GFC fraction with the highest antibacterial activity against multidrug-resistant *S. aureus* ATCC BAA-44 were collected for LCMS-IT-TOF and MS/MS analyses (Table 4).

### 4.11. Microbroth Susceptibility Assay of GPC Fractions against S. aureus ATCC BAA-44

Microbroth assay was performed to demonstrate the antibacterial activity of crude extract and its corresponding GPC fractions against the multidrug-resistant *S. aureus* ATCC BAA-44 strain [65]. Briefly, a bacterial cell suspension adjusted to 1.0 × 10^6^ cfu/mL in tryptic soy broth medium was prepared. Five μL of 200 mg/mL honey extract stock solution as well as the GPC fraction, 5 μL of 10 mg/mL tetracycline as the positive control, and 5 μL of DMSO as the negative control were placed in different wells (96-well plate) in triplicates. Afterwards, 195 μL of bacterial cell suspension was added to each well. Finally, 200 μL of tryptic soy broth was dispensed in wells that served as blanks. The plate was incubated at 37 °C and the optical density was measured at 620 nm after 18–24 h incubation using a microplate reader (Multiskan FC, Thermo Scientific, USA). All test pathogens in this study were not sensitive to DMSO (Table 2). The assay was performed in triplicates. Percent inhibition was measured relative to the untreated tested bacteria.

### 4.12. Determination of Minimum Inhibitory Concentration (MIC)

The minimum inhibitory concentration (MIC) of SL 01 honey crude extract against multidrug-resistant *S. aureus* ATCC BAA-44 was determined using broth microdilution method as previously described by Dalisay et al. (2013) [65]. Briefly, 2-fold serial dilutions of honey crude extracts in DMSO (200 mg/mL) stock solution were prepared in 96-well microtiter to yield a final test concentration range of 5000 to 9.8 μg/mL. Bacterial inoculum was prepared from 24 h culture on tryptic soy agar at 37 °C. The inoculum was diluted into Tryptic Soy broth to yield a final inoculum with an optical density of 1.0 × 10^6^ cfu/mL. The microdilution wells, which contained 5 μL of the serially diluted extracts, were inoculated with 195 μL of the resulting bacterial suspension. Tetracycline and DMSO were used as positive and negative controls, respectively. The inoculated plates were incubated at 37 °C for 18 h. Determinations were carried out in triplicates. The inhibition of growth was determined by measuring the OD at 600 nm. The MIC end point was defined as the lowest concentration with 90% growth inhibition.

### 4.13. Ultra High Pressure Liquid Chromatography Mass Spectrometry (UPLCMS) and MS/MS Analysis of Bioactive GPC Fraction

High-resolution MS and MS/MS experiment of the HPLC UV peak (λ_max_ 254 nm) of bioactive GPC (see Section 2.4) fraction were acquired using a Shimadzu LCMS-IT-TOF equipped with a Prominence HPLC system (SIL-20A HT autosampler, LC-20AD pump system, SDP-M20A diode array detector) with reversed-phase column (Phenomenex Luna C_8_ column, 5 μm, 150 × 2 mm) and a gradient of MeOH in H_2_O containing 0.01% formic acid as eluent (0–100% over 30 min) at a flow rate 0.5 mL/min. The sample load was 20 μL with a concentration of 2.5 mg/mL (50:50 MeOH:H_2_O). The MS analysis used the following conditions: block temperature—300 °C, DL temperature—250 °C, nebulizing gas flow rate—1.5 L/min, drying gas flow rate—15.0 L/min, mass range—110–900 amu (positive and negative mode), event time—100 ms, and the ion accumulation time—30 ms.

The MS/MS experiment was performed in 50% collision energy. The identified antibacterial compound was set as the precursor ion and fragments with mass range of *m*/*z* 50–900 in positive mode were recorded. The identity of the metabolites was obtained after comparison with the databases such as METLIN^TM^, AntiBase, PubChem, and ChemSpider. Fragmentation of the metabolite of interest was also generated for confirmation.

### 4.14. Data Processing and Statistical Analysis

The antibacterial and antioxidant activities were expressed as mean ± standard error. Paired *t*-test analysis was performed to determine significance in the mean values of antibacterial and antioxidant activities. The heat map that shows the antibacterial activity, antioxidant activity, flavonoid content, and phenolic content of Philippine honey and Manuka honey was constructed using TreeView 3.0 (http://jtreeview.sourceforge.net/, accessed on 17 January 2021) [66]. Data for each test were normalized and relative values were subjected to data correlation using City Block Distance with complete linkage calculation between groups.

## 5. Conclusions

The extracts from honey produced by the stingless bee *T. biroi* exhibited antibacterial activity against *Staphylococcal* pathogens with strong activity against the multidrug-resistant *S. aureus* and demonstrates high antioxidant activity compared to *A. mellifera* honey extracts. The flavonoids and phenolic compounds found in Philippine honey extracts have a direct link to the in vitro antibacterial activities as well as antioxidant activities. This is the first antibiotic activity screening of Philippine honey extracts produced by *A. mellifera* and *T. biroi* against *Staphylococcal* pathogens and antioxidant evaluation. This preliminary work showed isorhamnetin as one of the antibiotic components found in *T. biroi* honey extract. The results of this preliminary study increase our knowledge of Philippine honey as a potential nutraceutical agent with therapeutic benefits against *Staphylococcal* pathogens. Future studies using a broader set of Philippine *T. biroi* honey samples from various geographical areas, coupled with melissopalynological analysis for identification of the botanical origins, are needed to evaluate their therapeutic potential. Moreover, chemical profiling and metabolomics of the samples should be performed to investigate the metabolite composition that may serve as parameters to reveal biological activities, chemodiversity, as well as the geographical, entomological, and plant origins of Philippine honeys with therapeutic nutraceutical potential.

## Figures and Tables

**Table 1:** Floral sources, harvesting region, and honey bee species.

Honey Sample (Code)	Floral Source	Geographical Origin	Bee Species
MGO 550 ^a^	Manuka tree	New Zealand	*Apis mellifera*
UMF 15 ^a^	Manuka tree	New Zealand	*Apis mellifera*
NL 01	Sunflower	NL, Philippines	*Apis mellifera*
NL 02	Sunflower (mostly), trumpet flowers, squash, calliandra, coffee	NL, Philippines	*Apis mellifera*
NM 01	Wild flowers, falcata	NM, Philippines	*Apis mellifera*
NM 02	Cassava, minimal sunflowers	NM, Philippines	*Apis mellifera*
NM 03	Pineapples	NM, Philippines	*Apis mellifera*
NM 04	Pineapples	NM, Philippines	*Apis mellifera*
NM 05	Pineapples, minimal sunflowers	NM, Philippines	*Apis mellifera*
SEM 01	Banana trees, coconuts, palm trees, Philippine lime	SM, Philippines	*Apis mellifera*
SL 01	Coconut, bananas, mangoes	SL, Philippines	*Tetragonula biroi*
SL 02	Coconut, acacia, tamarind, sapodilla fruit, mangrove, kerson fruit	SL, Philippines	*Apis mellifera*
SL 03	Coconut, mahogany, mangoes, dragon fruit, bamboo, peanut grass, other flowering plants	SL, Philippines	*Apis mellifera*
SL 04	Acacia, mangoes, coconut, tamarind, avocadoes, coffee	SL, Philippines	*Apis mellifera*
SL 05	Kerson fruit, mangoes, coconuts, calamansi, mangroves, papaya	SL, Philippines	*Apis mellifera*
WV 01	Mahogany, Philippine lime, cucumber tree, yellow bell plant	WV, Philippines	*Apis mellifera*
WV 02	Banana, coconut, cosmos flower, nipa palm and mangoes	WV, Philippines	*Apis mellifera*

^a^ Manuka honey—used as a reference honey. NL—Northern Luzon; NM—Northern Mindanao; SL—Southern Luzon; SM—Southern Mindanao; WV—Western Visayas.

**Table 2:** Antibacterial Activity of Honey Extracts Against Staphylococcal Pathogens.

Honey Samples ^a^	Antibiotic Activity Zone of Inhibition, mm				
	*S. aureus* ATCC BAA-44	*S. aureus* ATCC 25923	*S. aureus* ATCC 6538	*S. saprophyticus* Clinical Isolate	*S. epidermidis* Clinical Isolate
Tetracycline (standard) ^b^	22.0 ± 0.7	26.5 ± 1.4	24 ± 0.0	33 ± 2.8	36.5 ± 2.8
DMSO ^c^	(-)	(-)	(-)	(-)	(-)
Manuka health MGO 550 ^d^	2.5 ± 0.7	(-)	(-)	6.0 ± 0.0	2.5 ± 0.7
Comvita UMF 15 ^d^	2.0 ± 0.0	(-)	(-)	5.8 ± 1.1	2.5 ± 0.7
NM 01	(-)	(-)	1.5 ± 2.1	4.0 ± 1.4	(-)
NM 02	(-)	(-)	(-)	4.5 ± 0.7	(-)
NM 03	(-)	(-)	(-)	3.5 ± 0.7	(-)
NM 04	(-)	(-)	(-)	3.5 ± 2.1	(-)
NM 05	(-)	(-)	(-)	2.5 ± 0.7	(-)
SL 01	12.5 ± 0.7	4.8 ± 0.4	3.5 ± 0.7	5.0 ± 1.4	4.5 ± 0.7
SL 02	(-)	(-)	(-)	3.0 ± 1.4	(-)
SL 03	(-)	(-)	(-)	5.0 ± 0.0	(-)
SL 04	(-)	(-)	(-)	5.0 ± 1.4	(-)
SL 05	(-)	(-)	(-)	3.3 ± 0.4	(-)
SEM 01	(-)	(-)	2.5 ± 0.7	2.5 ± 0.7	(-)
NL 01	(-)	(-)	(-)	4.3 ± 0.4	(-)
NL 02	(-)	(-)	(-)	(-)	(-)
WV 01	(-)	(-)	2.0 ± 0.0	(-)	(-)
WV 02	(-)	(-)	(-)	(-)	(-)
% Hit rate (Philippine honey *n* = 15)	7	7	27	80	7

^a^ Honey extract samples (10 mg/well); ^b^ Tetracycline (0.25 mg/well), positive control; ^c^ DMSO, negative control; ^d^ Manuka honey from New Zealand used as reference; (-)—no activity.

**Table 3:** Total Phenolic and Flavonoid Content of Honey Crude Extracts.

Honey Samples	μg GAE/mg ^a^	μg QE/mg ^b^
MGO 550	184.655 ± 5.226	21.857 ± 0.006
UMF 15	170.500 ± 15.144	26.857 ± 0.009
NL 01	204.872 ± 21.868	19.000 ± 0.016
NL 02	233.246 ± 3.918	8.762 ± 0.002
NM 01	200.402 ± 3.788	10.667 ± 0.003
NM 02	236.192 ± 1.371	6.857 ± 0.001
NM 03	180.955 ± 23.681	9.714 ± 0.001
NM 04	165.884 ± 0.222	7.333 ± 0.002
NM 05	235.739 ± 1.278	10.905 ± 0.005
SEM 01	224.221 ± 13.914	13.048 ± 0.001
SL 01	227.128 ± 11.064	216.143 ± 0.016
SL 02	224.809 ± 3.190	14.714 ± 0.003
SL 03	192.081 ± 5.431	12.571 ± 0.004
SL 04	229.846 ± 0.222	21.619 ± 0.004
SL 05	234.569 ± 3.063	9.952 ± 0.002
WV 01	192.973 ± 15.720	11.143 ± 0.002
WV 02	218.138 ± 3.909	8.048 ± 0.001

^a^ Gallic Acid Equivalent—unit for Total Phenolic Content per mg crude extract; ^b^ Quercetin Equivalent—unit for Total Flavonoids per mg crude extract.

**Table 4:** Gel Permeation Chromatography (GPC) Purification of SL 01 Honey Crude Extract Showing Eight Major Fractions, Color Profile and Yield and Antibiotic Activity against Multidrug-resistant *S. aureus* ATCC BAA-44.

Sample/Fraction	Visible Color Profile	Yield from 510.0 mg SL 01 Honey Crude Extract mg	% Growth Inhibition against Multidrug-Resistant *S. aureus* ATCC BAA-44
Tetracycline ^a^	-	-	99.2719 ± 0.03
DMSO ^b^	-	-	no activity
Crude extract	-	-	94.25 ± 0.24
1	colorless	5.1	no activity
2	red orange	23.5	no activity
3	yellow orange	149.5	no activity
4	green orange	219.2	72.5 ± 4.38 ^c^
5	yellow green	41.6	no activity
6	colorless	39.7	no activity
7	light yellow	24.1	82.1 ± 9.50 ^c^
8	light yellow green	7.1	no activity

^a^ Tetracycline (0.25 mg/mL); ^b^ DMSO; ^c^ Two-tailed *p* value equals 0.4109. Honey samples were tested at 5 mg/mL.

## Data Availability

The data presented in this study are available in the Supplementary Materials.

## Supplementary Materials

The following are available online. Figure S1: Philippine Map annotated with collection sites of honey samples. Figure S2: FTIR spectral features of honey crude extracts used for the quantification of phenolic acids in honey extracts. The sample FTIR spectrum shown was SL 01 honey. Figure S3: The root mean square error of prediction (RMSEP) of the partial least squares regression (PLSR) model with respect to the number of components. Savitzky Golay was applied for spectral smoothing of all honey samples used in this study. Three components were chosen with RMSEP = 0.08611 and R2 = 0.9926. Savitkzy Golay was performed, with a three-point filter and wavelet transform scale of three, to enhance the resolution of spectral features and to minimize problems from baseline shifts. Figure S4: The partial least squares (PLS) train model of three components used for the quantification of total phenolic acid content in honey samples in this study. The line graph is the total of seven spectra of gallic acid (99.3%, ChromaDex, Los Angeles, CA, USA) were used to establish the calibration model. A leave-one-out cross validation was performed to evaluate the prediction power of the model. Figure S5: MS/MS fragmentation of *m*/*z* 317.0635 [M + H]^+^ using 50% collision energy and detected using LCMS-IT-TOF, *m*/*z* range from 50 to 900 in positive mode. * *m*/*z* 318.0670 and *m*/*z* 319.06 (a very small peak) are residue isotopic peaks of precursor ion *m*/*z* 317.0635 [M + H]^+^. Figure S6: HPLC Chromatogram of SL 01 crude extract and GPC Fraction 5 showing peaks at λ_max_ 254 nm. Figure S7: HPLC Chromatogram of SL 01 crude extract and GPC Fraction 4 showing peaks at λ_max_ 254 nm.
