# Supplementary material for: The Isorhamnetin-Containing Fraction of Philippine Honey Produced by the Stingless Bee Tetragonula biroi Is an Antibiotic against Multidrug-Resistant Staphylococcus aureus
Source: Molecules. 2021 Mar 17;26(6):1688. doi: 10.3390/molecules26061688 (PMC8002709; doi:10.3390/molecules26061688)
Supplement: Supplementary file 1 [file molecules-26-01688-s001.zip › molecules-1097087-supplementary.pdf]

Supporting Information

The Isorhamnetin-Containing Fraction of Philippine Honey Produced by the Stingless Bee *Tetragonula biroi* is an Antibiotic Against Multidrug-Resistant *Staphylococcus aureus*

Angelica Faith L. Suarez [1], April Dawn G. Tirador [1], Zenith M. Villorente [2], Cathrina F. Bagarinao [2], Jan Vincent N. Sollesta [2], Gerard G. Dumancas [3,4], Zhe Sun [5], Zhao Qi Zhan [5], Jonel P. Saludes [4,6,7] and Doralyn S. Dalisay [1,4,8,*]

1 Center for Chemical Biology and Biotechnology (C2B2), University of San Agustin, Iloilo City 5000, Philippines; aicasuarez03@gmail.com (A.F.L.S.); tiradorapril@gmail.com (A.D.G.T.)

2 Maridan Industries, Inc., Jaro, Iloilo City 5000, Philippines; zmvillorente@maridan.com.ph (Z.M.V.); cfbagarinao@maridan.com.ph (C.F.B.); jvsollesta@maridan.com.ph (J.V.N.S.)

3 Department of Mathematics and Physical Sciences, Louisiana State University at Alexandria, Alexandria, 71302, LA, USA; gdumancas@lsua.edu (G.G.D.)

4 Balik Scientist Program, Philippine Council for Health Research and Development (PCHRD), Department of Science and Technology, Bicutan, Taguig City 1631, Philippines; jsaludes@usa.edu.ph

5 Shimadzu Asia Pacific (SAP), Singapore Science Park I, 118264, Singapore; sunzhe@shimadzu.com.sg (Z.S.); zhaoqi@shimadzu.com.sg (Z.Q.Z.)

6 Center for Natural Drug Discovery and Development (CND3), University of San Agustin, Iloilo City 5000, Philippines;

7 Department of Chemistry, College of Liberal Arts, Sciences, and Education, University of San Agustin, Iloilo City 5000, Philippines

8 Department of Biology, College of Liberal Arts, Sciences, and Education, University of San Agustin, Iloilo City 5000, Philippines

* Correspondence: ddalisay@usa.edu.ph; Tel.: +63-33-501-0350

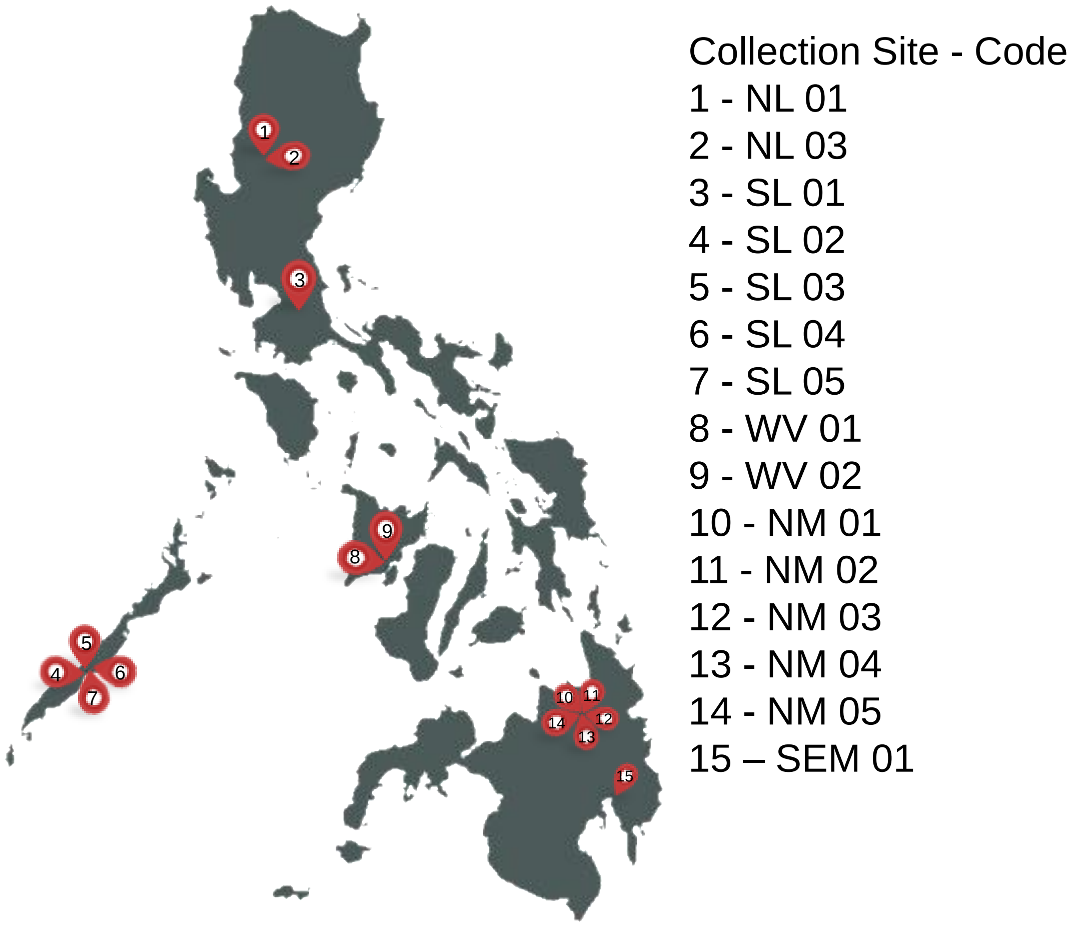

Figure S1. Philippine Map annotated with collection sites of honey samples.

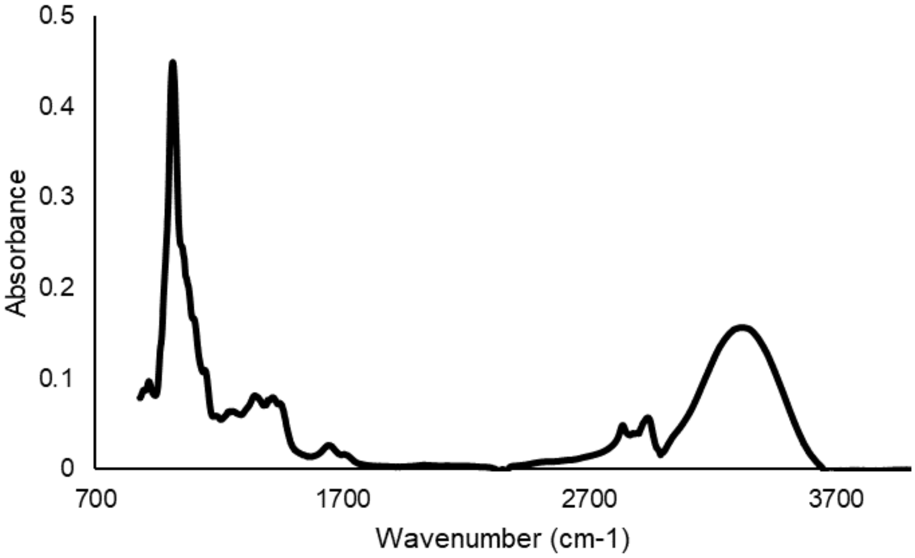

Figure S2. FTIR spectral features of honey crude extracts used for the quantification of phenolic acids in honey extracts. The sample FTIR spectrum shown was SL 01 honey.

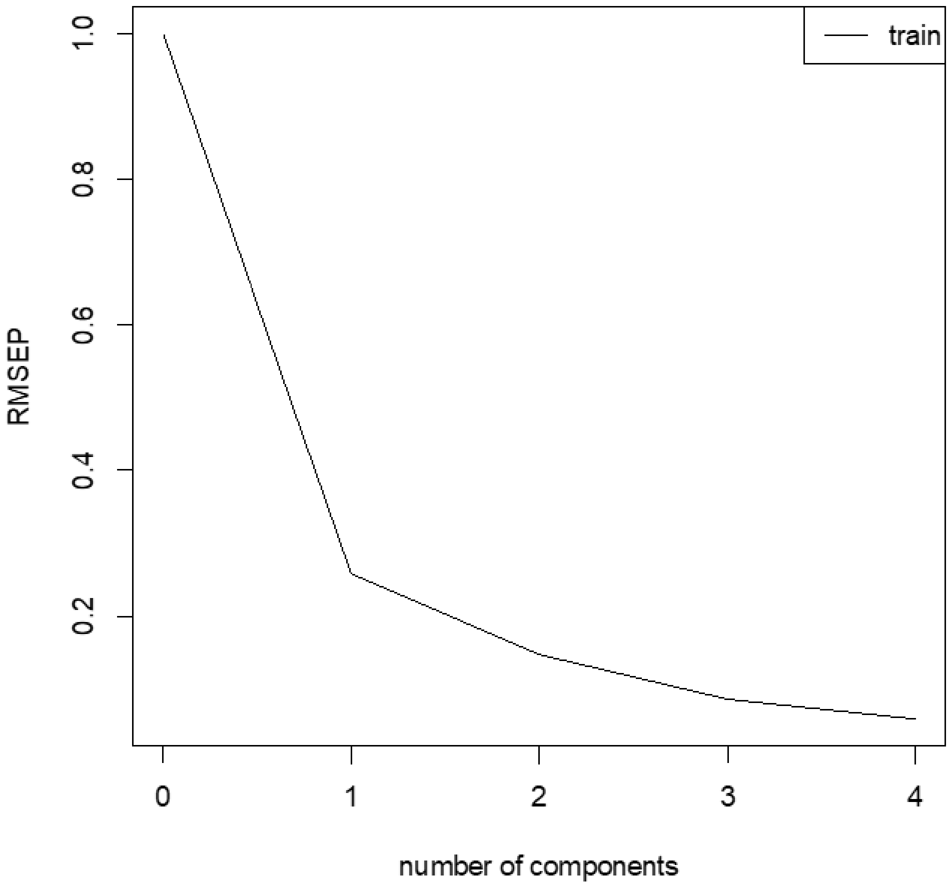

Figure S3. The root mean square error of prediction (RMSEP) of the partial least squares regression (PLSR) model with respect to the number of components. Savitzky Golay was applied for spectral smoothing of all honey samples used in this study. Three components were chosen with RMSEP = 0.08611 and R^2 = 0.9926. Savitkzy Golay was performed, with a three-point filter and wavelet transform scale of three, to enhance the resolution of spectral features and to minimize problems from baseline shifts.

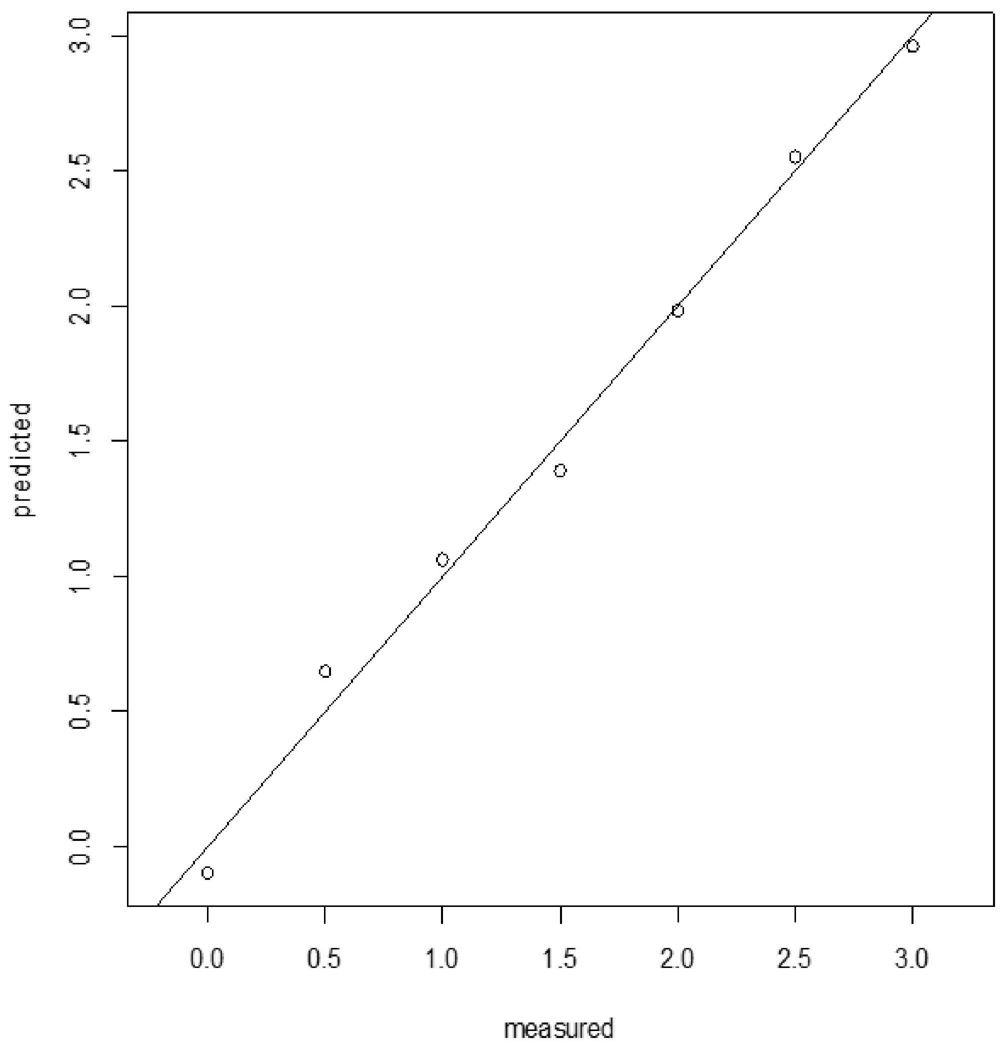

Figure S4. The partial least squares (PLS) train model of three components used for the quantification of total phenolic acid content in honey samples in this study. The line graph is the total of seven spectra of gallic acid (99.3%, ChromaDex, USA) used to establish the calibration model. A leave-one-out cross validation was performed to evaluate the prediction power of the model.

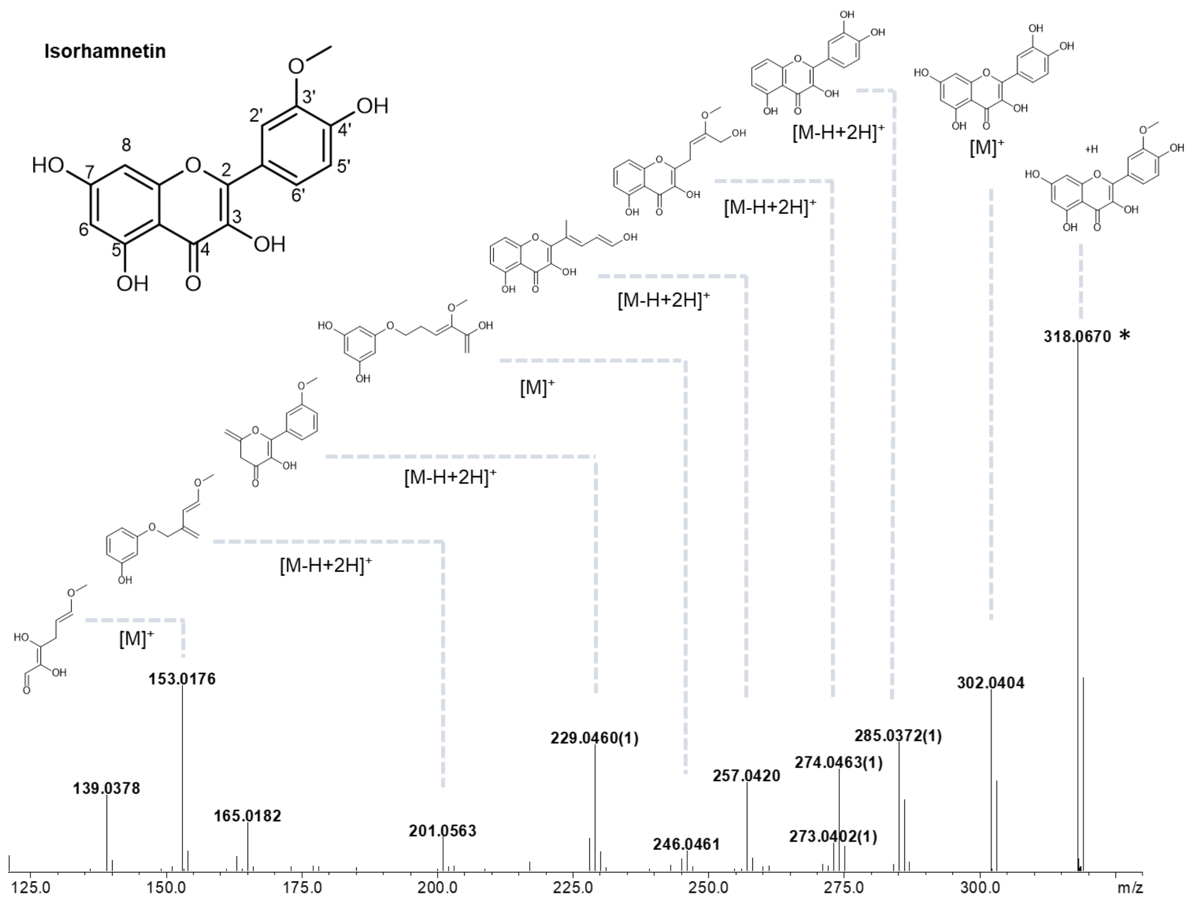

Figure S5. MS/MS fragmentation of *m/z* 317.0635 [M+H]+ using 50% collision energy and detected using LCMS-IT-TOF, *m/z* range from 50 to 900 in positive mode. **m/z* 318.0670 and *m/z* 319.06 (a very small peak) are residue isotopic peaks of precursor ion *m/z* 317.0635 [M+H]+.

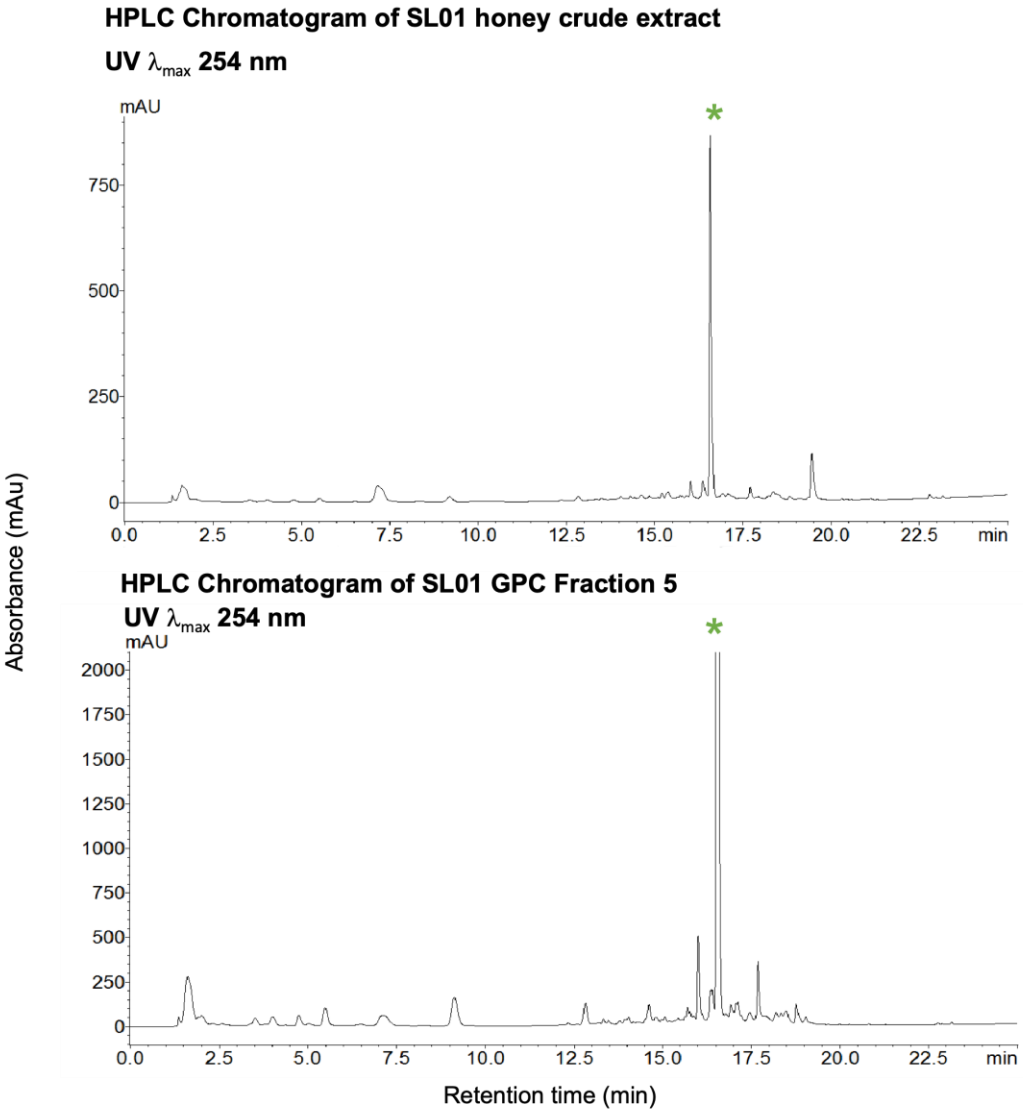

Figure S6: HPLC Chromatogram of SL 01 crude extract and GPC fraction 5 showing peaks at λ_{max} 254 nm.

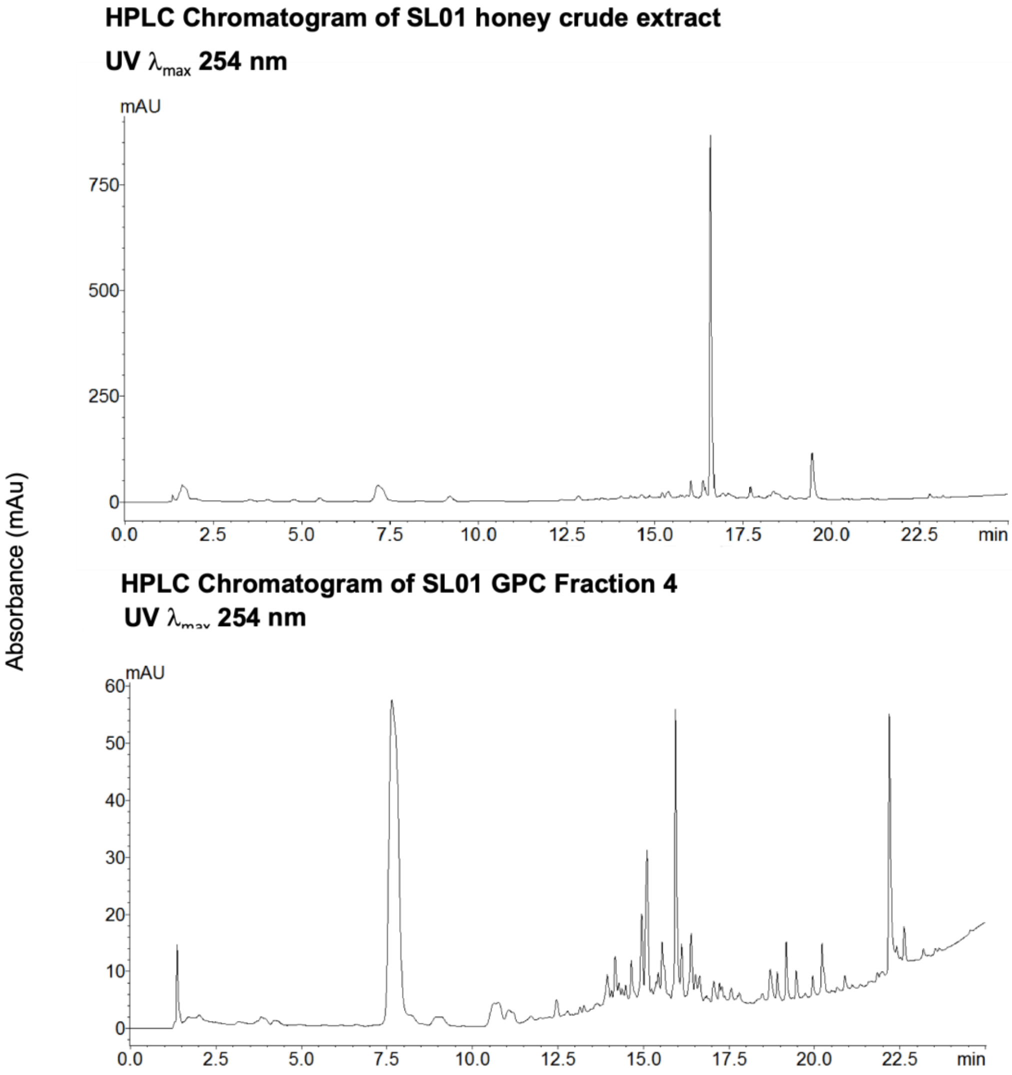

Figure S7: HPLC Chromatogram of SL 01 crude extract and GPC fraction 4 showing peaks at λ_{max} 254 nm.
